# Fully automated processor chip design: motivation, challenges and future directions

**DOI:** 10.1093/nsr/nwaf532

**Published:** 2025-11-29

**Authors:** Rui Zhang, Jiaming Guo, Shuyao Cheng, Yunji Chen

**Affiliations:** State Key Laboratory of Processors, Institute of Computing Technology, Chinese Academy of Sciences, China; State Key Laboratory of Processors, Institute of Computing Technology, Chinese Academy of Sciences, China; State Key Laboratory of Processors, Institute of Computing Technology, Chinese Academy of Sciences, China; State Key Laboratory of Processors, Institute of Computing Technology, Chinese Academy of Sciences, China

## Abstract

An overview of fully automated processor chip design, including its research motivations, three key challenges, and the overall framework with core components developed to address these challenges.

Processor chip design is a frontier research field that advances the progress of computer science. As the basic material carrier of information technology, processor chips power a variety of devices, including smartphones, personal computers, servers and Internet of Things devices.

To improve the efficiency of processor chip design, automated processor chip design is a key objective in the field of computer science. Unlike manual design, which relies on human effort to design and verify chips, the goal of automated processor chip design is to automate the design process and the verification process of processor chips. This issue originates from Church’s problem [1], proposed in 1957 by Alonzo Church: given inputs and outputs, how can a circuit be automatically designed to satisfy their relationships?

In the early days, electronic design automation (EDA) tools automated specific design steps, including logic synthesis, placement and routing, through predefined rules and Boolean logic. After that, optimization-based techniques were proposed to cope with the rapid increase in circuit complexity, such as design space exploration (DSE), which searches for superior design parameters to optimize the performance, power and area of the design. In recent years, AI techniques have introduced a more intelligent, data-driven era of processor chip design, substantially enhancing efficiency for complex designs. For example, neural networks are used to predict resource utilization and performance to improve DSE efficiency [2]; reinforcement learning (RL) is applied to logic optimization [3]; AlphaChip [4] uses RL and graph neural networks to achieve human-competitive placement; and conditional generative adversarial networks are used to predict routing congestion and guide routing optimization [5]. However, most of these AI-driven approaches serve only to refine individual steps within the conventional design flow. They have not fundamentally altered the overarching design paradigm and thus offer only incremental gains in overall automation and design performance.

## MOTIVATION

With the rapid development of information technology, the demand for processor chips has exploded. However, existing automation paradigms for processor chip design can no longer keep pace. Current automated design paradigms suffer from three fundamental limitations: constrained fabrication technology, limited resources and a diverse ecosystem. First, as semiconductor fabrication technologies advance toward the fundamental physical limits below the 7-nm node, existing automated design methods that rely on conventional performance scaling through fabrication technology become ineffective. Second, despite automation, conventional design flows still demand substantial expert involvement to design logic by coding in high-level programming languages or hardware description languages, resulting in protracted development cycles and substantial costs. Third, emerging AI, cloud and edge applications demand hardware-software co-optimization with a high degree of customization, which is beyond the capacity of current approaches that primarily use AI to refine isolated steps. Consequently, existing automated design paradigms fall short of delivering true end-to-end automation and optimal performance.

A new paradigm of fully automated processor chip design is urgently needed to overcome the aforementioned challenges. This paradigm automates both hardware and software development according to specified functional requirements. By leveraging emerging AI capabilities, such as large language models (LLMs), this approach can outperform conventional design flows under the same fabrication technology. Moreover, it minimizes manual intervention, greatly accelerates design cycles and reduces development costs. Furthermore, it supports rapid, application-specific customization of chip hardware and software stacks through custom design specification and constraints, meeting the growing demand for specialized computing solutions.

## CHALLENGES AND FUTURE DIRECTIONS

**Figure 1:**
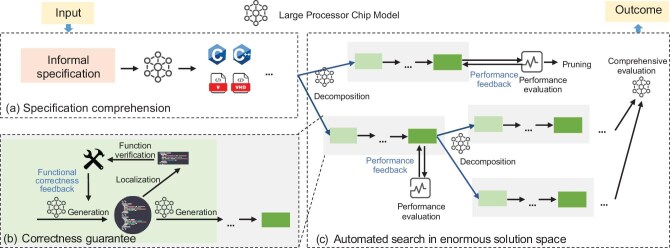
Potential framework for fully automated processor chip design, including three core components: (a) a domain-specific LLM to interpret the specification and generate a primary design; (b) an automated repair mechanism based on functional verification to guarantee the design’s correctness; (c) an automated search mechanism based on performance feedback to address the problem of an enormous solution space.

Recently, fully automated chip design has become a notable research topic. Several studies have made preliminary attempts to automate the design of circuits ranging from approximately 100 to 2500 gates by applying RL [6], random forests [7], ensemble learning [8] and LLM [9]. However, these approaches cannot meet the scale and precision requirements for complex circuits such as CPUs. To achieve fully automated processor chip design, three challenges need to be addressed: specification comprehension, correctness guarantee and an enormous solution space. To meet these challenges, a fully automated processor chip design system should integrate three core components, as illustrated in Fig. 1:

(1) A domain-specific LLM should be trained to comprehend the specification and generate a primary design.
(2) An automated repair mechanism based on functional verification is needed to guarantee the design’s correctness.
(3) An automated search mechanism based on performance feedback is required to address the problem of an enormous solution space.

The following sections present each of these challenges and elaborate on their corresponding core components and future research directions.

### Specification comprehension

In practice, the design specifications for processor chips are often conveyed in ambiguous, informal natural language, whereas existing automated design tools accept only precise, formal inputs, such as C/C++ code or hardware description languages like VHDL and Verilog. Converting informal requirements into these formal specifications still requires substantial manual intervention from experts. Consequently, a fully automated design paradigm must comprehend functional requirements in natural language, resolving inherent ambiguities and compensating for omissions. Currently, LLMs have demonstrated the capability to generate formal programming languages from informal natural language. They have shown the ability to automatically generate code based on natural language descriptions, ranging from short feature modules to longer whole applications. Although all-purpose LLMs exhibit an ability to comprehend certain specifications, domain-specific training is beneficial for achieving higher performance and reliability. This process involves systematically collecting processor design data and conducting targeted training. Nevertheless, training such models is constrained by data scarcity. A potential solution is to leverage LLMs themselves to perform automatic summarization and cross-verification of these artifacts, thereby constructing higher-quality training corpora at scale. For example, CodeV-R1 [10] proposes a round-trip data synthesis method to produce a high-quality reasoning RTL design corpus. When fine-tuned with the higher-quality RTL design corpus, the 7B CodeV-R1 demonstrates a strong capability to generate RTL code from natural language requirements, achieving performance comparable to—or even surpassing—that of the 671B DeepSeek-R1. Thus, training a domain-specific LLM is of significant importance in addressing the challenge of specification comprehension.

### Correctness guarantee

Processor chip design demands extremely high standards of correctness. For instance, the functional verification of Intel’s Pentium 4 processor required 99.99999999999% correctness [11]. However, a fundamental conflict exists between the deterministic certainty required for processor chip verification and the probabilistic foundations of LLMs. To guarantee design correctness, automated repair based on functional verification is essential. Specifically, the functional correctness of an intermediate design can be evaluated using specialized tools or models. When a functional error is detected, automatic repair is required; that is, the process falls back to the previous intermediate design whose correctness has been verified, and the current step is regenerated based on the error feedback of the current verification. The above verification and repair process is iterated continuously until the generated design passes the correctness verification. Through automated repair based on functional verification, design errors can be continuously found and corrected to ensure 100% functional correctness. The fully automated CPU, named Enlightenment-1 (also called QiMeng-CPU-v1) [12], was developed based on the concept of an automated repair mechanism. The circuit logic of QiMeng-CPU-v1 is represented using a novel graph structure, the binary speculation diagram (BSD), where functional verification is performed by calculating Boolean distance, and automated repair is accomplished through BSD expansion. QiMeng-CPU-v1 achieves over 99.99999999999% accuracy and can run Linux correctly, presenting a compelling case for addressing the correctness guarantee challenge.

### Enormous solution space

The design of a processor chip consists of several stages, from foundational software and logical design to circuit and physical design. Therefore, modeling the design space directly at the raw bitstream level and searching for the optimal solution within it leads to a dimensional explosion. For example, a 32-bit CPU possesses a solution space whose size is approximately $10^{10^{540}}$. Finding processor designs that are both functionally correct and performance-optimized within this enormous solution space presents significant challenges. To improve the performance and efficiency of fully automated design in the face of such a vast solution space, automated search based on performance feedback is necessary. In this process, the solution space is hierarchically decomposed to build a search tree. The performance prediction or actual performance evaluation of intermediate nodes is used as feedback to prune suboptimal search branches, thereby reducing the size of the solution space. Based on the current optimal search branch, subsequent optimization solutions can be generated. A similar idea has been successfully applied in automated foundational software design. For example, QiMeng-TensorOp [13] and QiMeng-Xpiler [14] leverage the idea of automated search based on performance feedback to generate high-performance tensor operators and transcompiling tensor programs, respectively. In these works, the solution space is modeled as a dynamic tree, and Monte Carlo tree search is leveraged to automatically discover the optimal solutions, with real execution time serving as performance feedback to guide exploration for high-performance solutions. Automated search based on performance feedback is expected to substantially reduce the size of the solution space and address the challenge posed by its enormous scale.

## DISCUSSION

Although the fully automated design framework introduces a new paradigm for processor chip design, it does not conflict with the conventional EDA flow. Instead, existing EDA tools can be integrated into the framework to accomplish some key steps, such as logic optimization, floorplan and placement, performance evaluation and functional verification. In practice, LLMs can call existing EDA tools by generating scripts to implement specific steps in the overall framework [9]. In conclusion, the fully automated design framework can be integrated with the established EDA ecosystem to further enhance overall design capabilities.
